# Comparison of Biomarkers of Oxidative Stress, Inflammation, and Endothelial Function Among Vegetarians and Non-vegetarians of Vijayapura, India: A Pilot Study

**DOI:** 10.7759/cureus.68342

**Published:** 2024-08-31

**Authors:** Muskan Page, Shrilaxmi Bagali, Kusal K Das

**Affiliations:** 1 Allied Health Science, BLDE (Deemed to be University), Vijayapura, IND; 2 Physiology, Shri B. M. Patil Medical College, Hospital and Research Centre, BLDE (Deemed to be University), Vijayapura, IND

**Keywords:** c-reactive protein, serum malondialdehyde, nitric oxide, high-sensitivity c-reactive protein, hs-crp, oxidative stress, no, mda, non-vegetarian, vegetarian

## Abstract

Background: The known impact of diet on the pathophysiology of various chronic diseases and the current dietary transition in the country make it essential to assess the influence of dietary patterns specific to this region on inflammation, oxidative stress, and endothelial functions.

Objective: This study compared oxidative stress, inflammation, and endothelial functions among vegetarians and non-vegetarians in Vijayapura, Karnataka, India.

Methods: The present cross-sectional comparative study involved apparently healthy vegetarians (n=35) and non-vegetarians (n=35) aged 20-40. The anthropometric measurements like height (cm) and weight (kg) were recorded, and the BMI was calculated. The physiological parameters like systolic and diastolic blood pressure and pulse rate were recorded. Serum high-sensitivity C-reactive protein (hs-CRP), serum malondialdehyde (MDA), and serum nitric oxide (NO) were estimated as markers of inflammation, oxidative stress, and endothelial function, respectively. Statistical analysis was done using SPSS Statistics version 20.0 (IBM Corp. Released 2011. IBM SPSS Statistics for Windows, Version 20.0. Armonk, NY: IBM Corp.).

Results: The average age of vegetarians and non-vegetarians was 25.22 ± 7.63 years and 25.60 ± 5.64 years, respectively. Anthropometric and physiological parameters were comparable between the two groups. However, there was a trend for higher mean body weight among non-vegetarians (53.94± 6.73 vs. 57.22±7.18) with a marginal non-statistically significant p-value (p=0.052). Vegetarians showed significantly higher serum MDA levels than non-vegetarians (2.14 (0.93-2.91) vs. 0.64 (0.35-1.32); p=0.000), while hs-CRP (vegetarians - 0.01 (0.005-0.034) vs. non-vegetarians - 0.03 (0.01-0.04); p=0.18) and serum NO levels (vegetarians - 6.72 (5.46-8.39) vs. non-vegetarians - 5.43 (2.87-9.16); p=0.215) were similar in both groups.

Conclusion: The results were intriguing and contrasting, as serum MDA is remarkably higher among vegetarians than non-vegetarians, pointing toward greater oxidative stress among the former and possibly indicating a dietary imbalance among vegetarians, which needs further exploration.

## Introduction

Oxidative stress arises when reactive oxygen species (ROS) outnumber antioxidants or radical scavengers [1]. Excess ROS can oxidize biomolecules or structurally alter proteins and genes, triggering inflammatory processes. These inflammatory processes intensify ROS production, aggravating oxidative stress and diminishing cellular antioxidant capacity. Thus, oxidative stress and inflammation are interconnected in a mutually reinforcing manner [2]. Additionally, oxidative stress has a role in endothelial cell activation, a crucial early stage in endothelial dysfunction [3]. Therefore, oxidative stress, inflammation, and endothelial dysfunction are interrelated components of an etiological network underlying the development of cardiovascular diseases [4].

The adage “You are what you eat” emphasizes the importance of diet/food in an individual’s overall health and well-being. Evidence suggests that food and its components, including nutrients and non-nutrient food components, influence acute and chronic inflammation [5].

In India, about a third of the population has been vegetarian since childhood, unlike in the West, where vegetarianism is adopted by choice in adulthood [6]. The traditional vegetarian diet in India consists of grains, legumes, pulses, nuts, vegetables, fruits, dairy, spices, and seasonings unique to the Indian diet [7]. The non-vegetarian diet in India consists of meat, chicken/fish/eggs, other seafood, and the foods vegetarians eat. Dietary patterns in India are further influenced by religion, culture and social identity, local agricultural practices, and the availability of diverse foods [8], leading to significant variations in diets across regions and states. Therefore, the impact of vegetarian and non-vegetarian dietary patterns on oxidative stress, inflammation, and endothelial function in Vijayapura, Karnataka, might produce findings that may differ from analogous studies conducted in Western countries.

The present study evaluated and compared oxidative stress, inflammation, and endothelial function among vegetarians and non-vegetarians in Vijayapura, India. We hypothesize that a vegetarian diet is associated with lower oxidative stress, inflammation, and better endothelial functions than a non-vegetarian diet.

## Materials and methods

Study design and population

This cross-sectional study involved 70 healthy subjects in Vijayapura aged 20-40 years from both genders. The study was conducted at the medical college hospital. Healthy attendants accompanying patients were conveniently sampled and included in the study. The study participants were interviewed regarding their dietary patterns and categorized into vegetarians and non-vegetarians based on the following operational definitions: vegetarians are "individuals who primarily consume plant-based foods such as fruits, vegetables, legumes, nuts, seeds, and grains and avoid meat, poultry, fish, and seafood, but still include dairy products in their diet (lacto-vegetarian)” [9]. Non-vegetarians are "individuals who consume meat, fish, eggs, chicken, and other seafood at least twice a week, along with plant-based foods (omnivores)” [9].

Calculation of sample size

G* Power version 3.1.9.4 software was used for sample size calculation. The anticipated mean±SD of serum malondialdehyde (MDA) in vegetarian and non-vegetarian individuals is 4.09±1.00 and 6.30±2.49, respectively [10]. The required minimum sample size is 34 per group (i.e., a total sample size of 68, assuming equal group sizes) to achieve a power of 95% and a significance level of 5% (two-sided) for detecting a true difference in means between two groups.

N=2[((Z∝+Zβ )*S)/d]^2)

Where Zα is the level of significance (95%), Zβ is the power of the study (95%), d is the clinically significant difference between two parameters, and SD is the common standard deviation.

Exclusion criteria

Subjects with a history of cardiovascular diseases, diabetes mellitus, current infection, chronic inflammatory diseases, history of (at least two weeks) infection, surgery, a trauma in the past two weeks, cancer, H/O smoking, alcohol consumption, tobacco chewing, consuming medications like glucocorticoids, nonsteroidal anti-inflammatory drugs, multivitamins and antioxidants, etc. known to affect inflammatory markers, and a recent change in the dietary patterns or habits were excluded from the study [11].

Ethical consideration

Written informed consent was obtained from the study participants before their enrolment. Prior approval for the study was obtained from the Institutional Ethical Committee of BLDE (approval number: (DU)/IEC/714/2022-23) following the guidelines outlined by the Indian Council of Medical Research (2017). The study adhered to the Declaration of Helsinki throughout its duration.

Data collection

The data was collected in the morning between 8.00 am and 10.00 am. The participants were interviewed for details of their dietary patterns and classified as either vegetarians or non-vegetarians as per the operational definitions. The study participants were subjected to physical anthropometry. The height (cm) and weight (kg) were measured using a stadiometer and weighing balance. BMI was calculated by dividing body weight (kg) by height (m) squared. The physiological parameters like systolic and diastolic blood pressure (mm Hg) and heart rate (beats/min) were recorded after a supine rest of 10 minutes.

Blood sampling and biochemical analysis

The participants’ blood was sampled after an overnight fast (10-12 hours) in plain tubes, allowed to clot for 15-30 minutes, and centrifuged at 2000 x g for 10 minutes to obtain serum. The serum was aliquoted and stored at -20°C until the time of analysis. It was used to estimate markers of inflammation, oxidative stress, and endothelial function. High-sensitivity C-reactive protein (hs-CRP) was estimated by ELISA as a marker of inflammation using the commercially available kit (CRP ELISA, Catalog No. CR375C CALBIOTECH). Oxidative stress was assessed by estimating serum MDA, a lipid peroxidation product, by the method of Buege and Aust [12]. Endothelial function was assessed by estimating serum nitric oxide (NO) by Greiss reaction [13].

Statistical analysis

SPSS Statistics version 20.0 (IBM Corp. Released 2011; IBM SPSS Statistics for Windows, Version 20.0; Armonk, NY: IBM Corp.) was used for statistical analysis. Data was checked for normal distribution using the Shapiro-Wilk test. Parametric data are presented as mean ± standard deviation (SD) and analyzed using Student’s t-test. Non-parametric data are presented as median and interquartile range and analyzed using the Mann-Whitney U test. P<0.05 is considered statistically significant.

## Results

Table 1 compares anthropometric parameters like height, weight, and BMI among vegetarians and non-vegetarians. Both groups were similar in age. For the body weight, the trend indicates that non-vegetarians had a slightly higher mean weight compared to vegetarians. However, this difference was found to be marginally non-statistically significant, with a t-value of -1.974 and a p-value of 0.052. There were no significant differences in height and BMI between vegetarians and non-vegetarians.

**Table 1:** Anthropometric parameters among vegetarians and non-vegetarians Data are presented as mean ± SD; p<0.05 is considered significant; BMI: body mass index

	Vegetarian (n=35)	Non-vegetarian (n=35)	t	p-value
Male/female	19/16	16/19		
Age (years)	25.22 ± 7.63	25.60 ± 5.64	-0.232	0.818
Height (cm)	165.80 ± 6.81	167.85 ± 7.45	-1.196	0.236
Weight (kg)	53.94 ± 6.73	57.22 ± 7.18	-1.974	0.052
BMI (kg/m^2^)	19.57 ± 1.77	20.28 ± 1.93	-1.597	0.115

Table 2 displays a comparison of physiological parameters between vegetarians and non-vegetarians. The heart rate and systolic and diastolic blood pressures showed no significant differences between vegetarians and non-vegetarians.

**Table 2:** Physiological parameters among vegetarians and non-vegetarians Data are presented as mean ± SD; p<0.05 is considered significant; SBP: systolic blood pressure, DBP: diastolic blood pressure

	Vegetarian (n=35)	Non-vegetarian (n=35)	t	p-value
Heart rate (beats/min)	88.34 ± 7.09	88.20 ± 7.39	0.083	0.934
SBP (mm Hg)	112.00 ± 7.95	111.51 ± 7.65	0.260	0.795
DBP (mm Hg)	77.65 ± 5.87	78.47 ± 5.582	-0.327	0.661

Table 3 compares hs-CRP (inflammatory marker), serum MDA (a marker of oxidative stress), and serum NO (endothelial function marker) among vegetarians and non-vegetarians. Vegetarians had higher serum MDA compared to non-vegetarians. Serum hs-CRP and serum NO did not differ between the two groups.

**Table 3:** Comparison of markers of oxidative stress, inflammation, and endothelial function in vegetarians and non-vegetarians Data are presented as the median and interquartile range (Q1-Q3); *p<0.05 is considered significant; MDA: malondialdehyde, hs-CRP: high-sensitivity C-reactive protein, NO: nitric oxide

	Vegetarian (n=35)	Non-vegetarian (n=35)	Z	p-value
Serum MDA (µmol/L)	2.14 (0.93-2.91)	0.64 (0.35-1.32)	-4.00	0.000*
hs-CRP (mg/L)	0.01 (0.005-0.034)	0.03 (0.01-0.04)	-1.327	0.18
Serum NO (mmol/L)	6.72 (5.46-8.39)	5.43 (2.87 -9.16)	-1.240	0.215

## Discussion

The current research examined oxidative stress, inflammation, and endothelial function biomarkers in individuals on vegetarian and non-vegetarian diets in Vijayapura, India. The anthropometric and physiological parameters showed no significant differences between vegetarians and non-vegetarians.

Serum MDA was measured to evaluate oxidative stress. Notably, we observed elevated serum MDA levels in vegetarians. This observation contradicts most previous studies that reported lower serum MDA and lower oxidative stress in vegetarians compared to their non-vegetarian counterparts [10,14,15].

Ideally, a well-balanced vegetarian diet should encompass a variety of antioxidant-rich foods, such as fruits, vegetables, whole grains, soy, legumes, and nuts, that counter the generated free radicals [16]. Nevertheless, findings from the current study, gathered through informal interviews conducted by the investigator during data collection, indicate that vegetarians’ diets lacked sufficient quantities of vegetables and fruits. Instead, they predominantly consumed refined grains like white rice and preferred fried/fast and processed foods over traditional, wholesome options. This may be considered a form of "contaminated vegetarianism." The word "contaminated vegetarianism" describes a diet high in saturated fats, trans fats, refined sugar, full-fat dairy products, sweets, curries, and fried foods. Additionally, there was a general lack of awareness of the importance and benefits of traditional foods [17]. In the present study, informal interviews of the non-vegetarians revealed better food diversity. Since meat is expensive, non-vegetarians consume it infrequently [17]. In the present study, apart from meat, the non-vegetarian diet also included vegetables, fruits, legumes, and dairy, although the study has not quantified the intake. A study conducted in five cities in India with a significant number of participants from households revealed that the average intake of fruits and vegetables fell below the World Health Organization’s recommended amount of 400 grams per day for all income brackets [18]. A similar scenario may also be prevalent among vegetarians in this part of the country, which needs further evaluation.

Diets, especially those rich in fats or carbohydrates, are associated with oxidative stress by enhancing protein carbonylation and lipid peroxidation and reducing antioxidant defense. Given that the conventional Indian vegetarian diet is characterized by high carbohydrate content, it could contribute to oxidative stress, potentially explaining the heightened serum MDA levels observed in vegetarians [19]. Studies have noted that vegetarians have a lower antioxidant defense. Due to the lower quality and amount of protein found in vegetarian diets, there is an inadequate intake of amino acids containing sulfur, which may impact the concentration of antioxidants, particularly glutathione (GSH). Furthermore, it has been shown that vegetarians have lower amounts of gamma-glutamyl transferase, GSH, uric acid, and antioxidant micronutrients (zinc, copper, selenium, and iron). These factors also contribute to the reduced antioxidant defense in vegetarians [20]. Hence, an interplay of multiple factors like insufficient intake of fruits and vegetables, diets rich in fats or carbohydrates, lower concentrations of antioxidants, and deficiency of micronutrients may be responsible for the higher oxidative stress observed among vegetarians of this study, each one of which needs further evaluation. There are reports of non-vegetarians having higher levels of exogenous antioxidants (notably vitamin B12 and vitamin D) and antioxidant micronutrients like zinc, copper, selenium, and iron in contrast to vegetarians. These factors could have favored lower oxidative stress among non-vegetarians in this study.

C-reactive protein (CRP) is an acute-phase protein, an inflammation biomarker. High-sensitivity CRP enables the detection of subclinical inflammation and has demonstrated its effectiveness as one of the most potent indicators for predicting future cardiovascular events [21]. This research showed no disparity in hs-CRP levels between vegetarians and non-vegetarians. This finding aligns with previous studies that noted the absence of substantial differences in CRP levels between individuals adhering to vegetarian and non-vegetarian diets [22,23]. Some studies have documented reduced CRP levels in vegetarians when compared to those following non-vegetarian diets [24-26]. Interestingly, Ashraf et al. reported higher serum markers of inflammation like interleukin 6 (IL-6), interleukin 1 (IL-1), resistin, and CRP in vegetarians, including higher BMI, blood glucose, and serum insulin levels [27]. Likewise, a study reported higher proinflammatory markers (tumor necrosis factor α, IL-6, IL-1β, hs-CRP, and serum resistin) and lower antiinflammatory markers (IL-10 and adiponectin) among polycystic ovarian syndrome women on an Indian vegetarian diet compared to age and BMI-matched non-vegetarian counterparts [11].

Among various indicators of endothelial function, serum NO has been utilized as a biomarker [21]. It plays a vital role in maintaining vascular tone and reactivity. In the human body, NO is synthesized through the l-arginine-NO synthase (NOS) pathway [28]. Besides this endogenous NO generation, it is also produced through the NOS-independent nitrate-nitrite-NO pathway [29]. Nitrate (NO^3−^) in vegetables can be a physiological substrate for NO production. Dietary NO^3−^ is abundant in green leafy vegetables like rocket, green spinach, basil, radish, Swiss chard and bok choy, and red beetroot.

In this study, serum NO was comparable between vegetarians and non-vegetarians. Various clinical interventions utilizing NO^3-^-rich vegetables have improved metabolic and cardiovascular functions. These interventions achieved elevated NO levels, contributing to enhanced endothelial function, reduced blood pressure, and decreased arterial stiffness [28]. Most published studies exploring the influence of dietary elements on NO have concentrated on individual nutrients. Nevertheless, dietary intake typically involves a variety of foods in various combinations and proportions, forming complete diets or dietary patterns. There is a lack of information regarding the impact of diverse dietary patterns, particularly those associated with vegetarian and non-vegetarian diets, on NO synthesis and, consequently, serum levels. Future research should address how various diet patterns influence serum NO and the molecular pathways that underlie these effects [30].

Strengths, limitations, and recommendations

The study's strength is its inclusion of vegetarians and non-vegetarians from Vijayapura, representing locally specific food staples. Participants have been following either a vegetarian or non-vegetarian diet since childhood, allowing for an assessment of long-term dietary patterns. However, the study involved a small sample size, which limits the generalizability of the results as small samples barely represent the population. Therefore, the results need to be confirmed on a larger sample. Diets high in fruits and vegetables are rich in antioxidants and can reduce oxidative stress. However, the study did not assess the precise intake of fruits and vegetables and, therefore, cannot comment on the adequacy of antioxidant substances obtained through the diets. Consequently, it is unclear if the higher oxidative stress observed in vegetarians is due to insufficient intake of antioxidant substances in the food. A detailed dietary analysis involving micronutrients and macronutrients, assessing both the quality and quantity, needs to be done. Future studies should examine lifestyle factors such as sedentary behavior, physical activity, sleep, and stress, as these could potentially confound results related to oxidative stress. Oxidative stress results from an overproduction of free radicals or a deficient antioxidant defense; assessing levels of endogenous antioxidants and the influence of various dietary patterns on their levels will give a clearer picture of oxidant-antioxidant balance. While serum MDA is commonly used as a biomarker for oxidative stress, it has limitations, such as low stability in biological samples and rapid enzymatic degradation. Therefore, future studies can assess additional biomarkers of oxidative stress, such as isoprostanes, protein carbonyls, and 3-nitrotyrosine, among others.

## Conclusions

The findings were intriguing, as serum MDA, a biomarker of oxidative stress, exhibited significantly higher values in vegetarians than non-vegetarians in Vijayapura, Karnataka, India. This observation suggests greater oxidative stress due to a potential dietary imbalance among vegetarians. The present study involved a small sample size. The results of the study warrant further exploration with a large sample size and detailed dietary analysis.
